# Local Renin-Angiotensin System in Normal Hematopoietic and Multiple Myeloma-Related Progenitor Cells

**DOI:** 10.4274/tjh.2013.0011

**Published:** 2014-06-10

**Authors:** Burak Uz, Suzin Çatal Tatonyan, Müge Sayitoğlu, Yücel Erbilgin, Özden Hatırnaz, Salih Aksu, Yahya Büyükaşık, Nilgün Sayınalp, Hakan Göker, Osman İ. Özcebe, Uğur Özbek, İbrahim C. Haznedaroğlu

**Affiliations:** 1 Hacettepe University Faculty of Medicine, Department of Internal Medicine, Division of Hematology, Ankara, Turkey; 2 İstanbul University, Institute for Experimental Medicine Research, Department of Genetics, İstanbul, Turkey

**Keywords:** Renin-angiotensin system, Multiple myeloma, Progenitor cell, CD34+

## Abstract

**Objective:** The prominent functions of the local renin-angiotensin system (RAS) in primitive hematopoiesis further support the hypothesis that local autocrine bone marrow RAS could also be active in neoplastic hematopoiesis. The aim of this study is to examine critical RAS elements in normal CD34+ hematopoietic stem cells and multiple myeloma (MM)-related progenitor cells.

**Materials and Methods:** The study group comprised the total bone marrow cells (CBM) of 10 hematologically normal people, the CD34+ stem cell samples (CD34+CBM) of 9 healthy donors for allogeneic peripheral stem cell transplantation, and the CD34+ stem cell samples (CD34+MM) of 9 MM patients undergoing autologous peripheral stem cell transplantation. We searched for the gene expression of the major RAS components in healthy hematopoietic cells and myeloma cells by quantitative real-time polymerase chain reaction analysis.

**Results:** RENIN, angiotensinogen (ANGTS), and angiotensin converting enzyme-I (ACE I) mRNA expression levels of CBM were significantly higher than those in myeloma patients (p=0.03, p=0.002, and p=0.0008, respectively). Moreover, RENIN and ANGTS mRNA expression levels were significantly higher in CD34+ stem cell samples of healthy allogeneic donors compared to those in myeloma patients (p=0.001 and p=0.01). However, ACE I expression levels were similar in CD34+CBM and CD34+MM hematopoietic cells (p=0.89).

**Conclusion:** Although found to be lower than in the CBM and CD34+CBM hematopoietic cells, the local RAS components were also expressed in CD34+MM hematopoietic cells. This point should be kept in mind while focusing on the immunobiology of MM and the processing of autologous cells during the formation of transplantation treatment protocols.

## Abstract (tr)

**Amaç:** Lokal renin-anjiotensin sisteminin (RAS) primitif hematopoezde önemli fonksiyonlara sahip olması, lokal otokrin kemik iliği (Kİ) RAS’ının da neoplastik hematopoezde aktif olabileceği hipotezini desteklemektedir. Bu çalışmanın amacı normal CD34+ hematopoetik kök hücreler ve multipl miyelom (MM) ilişkili progenitor hücrelerde kritik RAS elemanlarını araştırmaktır.

**Gereç ve Yöntemler:** Çalışma grupları; hematolojik açıdan normal insanların toplam Kİ hücreleri (CBM) (n=10), allojeneik periferik kök hücre transplantasyonu için sağlıklı donörlerin CD34+ kök hücre örnekleri (CD34+CBM) (n=9), ve otolog periferik kök hücre transplantasyonu uygulanan MM hastalarının CD34+ kök hücre örnekleri (CD34+MM) (n=9) tarafından oluşturuldu. Bu örneklerde ana RAS elemanlarının gen ekspresyonları qRT-PCR yöntemiyle araştırıldı.

**Bulgular:** CBM grubunun RENIN, anjiotensinojen (ANGTS), ve anjiotensin dönüştürücü enzim-I (ACE I) mRNA ekspresyon düzeyleri miyelom hastalarından anlamlı olarak yüksek idi (sırasıyla, p=0,03, p=0,002, ve p=0,0008). Sağlıklı allojeneik donörlerin CD34+ kök hücre örneklerindeki RENIN ve ANGTS mRNA ekspresyon düzeyleri myelom hastalarının kök hücre örneklerinden anlamlı derecede yüksek bulundu (p=0,001 and p=0,01). Bununla birlikte, CD34+CBM ve CD34+MM hematopoetik hücrelerde ACE I ekspresyon düzeyleri arasında anlamlı fark gözlenmedi (p=0,89).

**Sonuç:** Lokal RAS elemanları, CBM ve CD34+CBM hematopoetik hücrelerinden daha az olmak kaydıyla, CD34+MM hematopoetik hücrelerinden de eksprese edilmektedir. Bu nokta MM’nin immünobiyolojisine odaklanmada ve transplantasyon tedavi protokollerinin oluşturulması sırasında otolog hücrelerin hazırlanmasında akılda tutulmalıdır.

## INTRODUCTION

The local hematopoietic renin-angiotensin system (RAS) affects the essential steps of hematopoiesis in the bone marrow (BM) microenvironment [1,2,3,4]. Myelopoiesis, erythropoiesis, thrombopoiesis, and other cellular lineages are influenced by the actions of the local BM RAS [3]. Besides those cellular effects, the local RAS [4,5] is also active in the BM stromal niche for the crucial governing of hematopoietic functions [6,7]. The local BM RAS affects hematopoiesis via both altering the internal signals of transcription factors regulating gene expressions and mediating the external signals from the growth factors secreted from the BM microenvironmental hematopoietic and stromal cells [8,9,10,11]. 

The RAS affects numerous biological events that are important for the formation and function of blood cells. Apoptosis, cellular proliferation, intracellular signaling, mobilization, angiogenesis, fibrosis within the cytokine network, and many other essential pathobiological events are affected by the critical RAS molecules [3,4,5,12,13]. Malignant blood cells, including multiple myeloma (MM) cells, are derived from the clonal neoplastic stem cells within a complex series of pathological proliferative steps. The local BM RAS could affect neoplastic tumoral blood cell production [14,15,16,17,18,19,20,21,22]. The local RAS is effective even in embryonic hematopoiesis [23,24,25,26]. The prominent functions of the local RAS in primitive hematopoiesis further support the hypothesis that the local autocrine BM RAS could also be active in neoplastic hematopoiesis [3].

The aim of this study is to examine critical RAS elements in normal CD34+ hematopoietic stem cells and MM-related progenitor cells. For this purpose, CD34+ hematopoietic cells obtained from healthy peripheral allogeneic hematopoietic stem cell transplantation donors and from MM patients undergoing peripheral autologous stem cell transplantation were analyzed via quantitative real-time polymerase chain reaction analysis (qRT-PCR). Normal BM cells obtained from hematologically normal people were also studied to detect the impact of precursor cell compartments on RAS expressions. Elucidation of the status of the local RAS molecules in early and neoplastic hematopoiesis represents a clinically relevant basic research area for better understanding of the biology of the diseases [4,5].

## MATERIALS AND METHODS

**Study Population**

The study group comprised the total bone marrow cells (CBM) of 10 normal people, the CD34+ stem cell samples (CD34+CBM) of 9 healthy donors for allogeneic peripheral stem cell transplantation, and the CD34+ stem cell samples (CD34+MM) of 9 MM patients undergoing autologous peripheral stem cell transplantation. The diagnoses of MM were reached based on the criteria of the International Myeloma Working Group [27]. At the time of the sample collection, all of the patients were in good health and well hydrated. Written informed consent was obtained from all participants and the study protocol was approved by the local ethics committee of Hacettepe University.

**Isolation of RNA and Synthesis of cDNA**

Stem cell samples were collected in 2 mL ethylenediaminetetraacetic acid tubes. Total RNA was isolated according to the manufacturer’s instructions (QIAGEN, Germany). RNA quality was measured by spectrophotometer (ND-1000, NanoDrop Technologies, Inc., USA), and 1 µg of total RNA was used. Random primers (20 µM, Roche Diagnostics, Germany), 10 mM dNTP set (Fermentas UAB, Lithuania), RiboLock RNase Inhibitor (20 U/µL, Fermentas) and Moloney murine leukemia virus reverse transcriptase (200 U/µL, Fermentas) were used for cDNA synthesis. cDNA samples were stored at -20 °C.

**Quantitative Real-Time Polymerase Chain Reaction Analysis**

We searched for the gene expression of the major RAS components including RENIN, angiotensinogen (ANGTS), angiotensin converting enzyme-I (ACE I), ACE II, angiotensin receptor-I (AGTR I), and AGTR II in healthy hematopoietic cells and myeloma cells by qRT-PCR. Previously designed primer-probes were used [28]. mRNA levels were normalized to CYPA and B-ACTIN genes.

The qRT-PCR analyses were performed using a LightCycler 480 instrument (Roche Diagnostics). Real-time amplification was performed with a final reaction mixture of 20 µL containing 5 µM of each primer, 0.5 µM of each probe, LightCycler 480 Probe Master Mix, and 100 ng/µL of cDNA. Each sample was studied in duplicate and all runs were repeated twice. The PCR protocol was as follows: initial denaturation at 95 °C for 7 min, and amplification segment at 5 s at 95 °C, 10 s at 60 °C, and 10 s at 72 °C for 45 cycles. The 2-Ct method was used to calculate relative expression levels determined from the qRT-PCR experiments [29] and results were given as percentages.

**Statistical**

SPSS 15.0 (SPSS Inc., USA) was used for all statistical analyses. The results were given as mean ± standard error for the data with normal distribution, as median (min-max) for the data without a normal distribution, and as ratio for the nominal data. Data distribution was tested with the Kolmogorov-Smirnov test. Homogeneities of variances were evaluated with Levene’s test. Nonparametric tests were used since the sample sizes of the groups were small. Differences between 2 groups were assessed by the Mann-Whitney U test. A p-value of ≤0.05 was considered statistically significant.

## RESULTS

**Patient Characteristics**

Five female and 4 male myeloma patients with a median age of 52 (40-62) years were recruited. Based on the International Staging System, 4 had stage I, 2 had stage II, and 2 had stage III disease. One patient could not be evaluated due to the lack of initial data. 

**Relative mRNA Expressions of the RAS Components in the Studied Cellular Samples**

**Figure 1:**
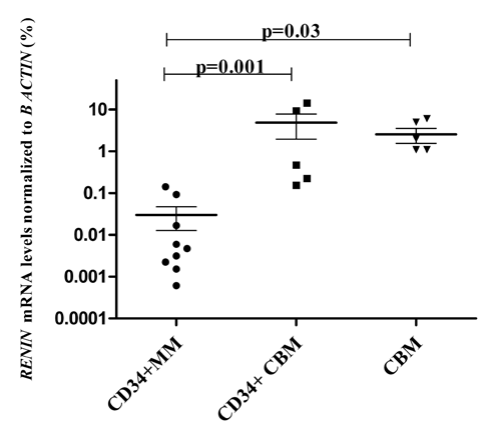
Relative RENIN mRNA expression levels (%) of total bone marrow cells of hematologically normal people (CBM), CD34+ stem cells of healthy allogeneic donors (CD34+CBM), and CD34+ stem cells of myeloma patients (CD34+MM).

**Figure 2:**
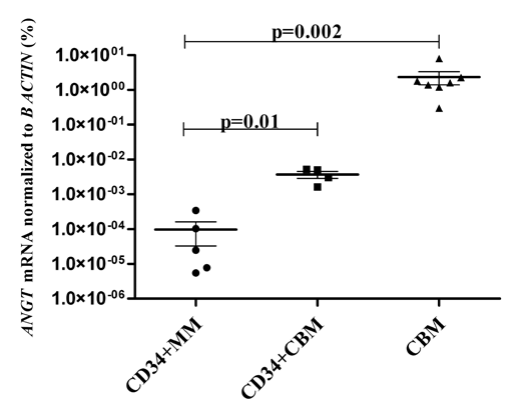
Relative ANGTS mRNA expression levels (%) of the total bone marrow cells of hematologically normal people (CBM), CD34+ stem cells of healthy allogeneic donors (CD34+CBM), and CD34+ stem cells of myeloma patients (CD34+MM).

**Figure 3:**
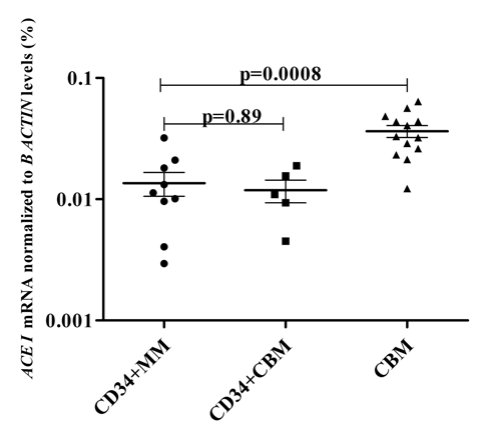
Relative ACE I mRNA expression levels (%) of the total bone marrow cells of hematologically normal people (CBM), CD34+ stem cells of healthy allogeneic donors (CD34+CBM), and CD34+ stem cells of myeloma patients (CD34+MM).

RENIN, ANGTS, and ACE I mRNA expression levels of CBM were significantly higher than those in myeloma patients (p=0.03, p=0.002, and p=0.0008, respectively; Figures 1, 2, 3). Moreover, RENIN and ANGTS mRNA expression levels were significantly higher in CD34+ stem cell samples of healthy allogeneic donors compared to those in myeloma patients (p=0.001 and p=0.01; Figures 1 and 2). However, ACE I expression levels were similar in the CD34+CBM and CD34+MM groups (p=0.89; Figure 3). Relative expression levels of RENIN, ANGTS, and ACE I genes in CD34+MM cells compared to CD34+CBM cells are given in Table 1. Other RAS pathway members’ (ACE II, AGTR I, and AGTR II) expressions were also examined and were not found to be at detectable levels, and no significant differences were determined between any groups. 

## DISCUSSION

In the present study, RENIN and ANGTS mRNA expressions were significantly higher in CD34+ hematopoietic stem cells of healthy allogeneic donors in comparison to myeloma-related progenitor cells. Likewise, RENIN, ANGTS, and ACE I mRNA expression levels of CBM were significantly higher than those in the myeloma patients. However, ACE I expression levels were similar in CD34+CBM and CD34+MM hematopoietic cells. These findings support our original hypothesis that there is a biologically active local RAS in the hematopoietic system in normal and pathological states [1,2].

Strawn et al. previously verified in rats that all of the main RAS components, including renin, ANGTS, ACE, and AGTR I, are detectable in the normal rat BM cellular compartment and rat microenvironmental stroma at the molecular and protein levels [6]. In the present study, we have found RENIN, ANGTS, and ACE I mRNA expressions in CD34+ stem cell samples of normal human subjects. ACE/CD143 was implicated in enhancing the recruitment of primitive stem cells into the S-phase by degrading AcSDKP [9,30,31,32,33]. ACE, converting Ang-I into Ang-II, is an important peptide for almost all aspects of hematopoiesis [23,31,34,35,36]. Myelopoietic effects of ACE and Ang-II are evident at the hematopoietic stem cell level, extending to the committed myeloid and erythroid lineages [35]. Hence, the local hematopoietic RAS seems to be effective in all species for cellular development. Hematopoiesis [37], myelopoiesis [35], erythropoiesis [38], thrombopoiesis [9], and other cellular lineages [37,39,40,41] are regulated by the actions of the peptides of the local BM RAS. The local BM RAS mediates those complicated networks of BM hematopoiesis in an autocrine/paracrine/intracrine fashion. The growth, production, proliferation and differentiation of the blood cells are affected by the hematopoietic RAS [1,4,5].

The local RAS is also expressed in the cellular compartment of the immunohematological system. The RAS is active in the production and function of distinct blood cell lineages such as dendritic cells, mast cells, T lymphocytes, monocytes, macrophages, and antigen-presenting cells [4,5]. For instance, ACE degrades substance P present in the BM microenvironment, lymphoblasts, and lymphocytes by cleaving a C-terminal dipeptide or tripeptide [41]. Stegbauer et al. reported the up-regulation of renin, ACE, and AGTR I in the immune system, including antigen-presenting cells in myelin-oligodendrocyte glycoprotein-induced experimental autoimmune diseases [42]. In this study, CD34+MM hematopoietic cells also locally expressed RENIN, ANGTS, and ACE I mRNA, indicating the activity of RAS in myeloma-related progenitor cells. ACE activity was also linked to MM [43,44]. BM AGTR I expression levels of myeloma patients showed a positive correlation with their BM infiltration pattern and tumor load, indicated by serum β2 microglobulin levels [44]. Our results about myeloma-related progenitors in this study provide an additional clue for the local RAS effects in the pathobiology of MM.

Recent studies [23,24,25,26] focused on the status of ACE within the context of the local RAS, the earliest human embryonic hematopoietic stem cells [45], and the developmental sequence underlying the ontogeny of human blood cells. The local RAS regulates the genesis and function of the hematopoietic system starting from embryonic life [25]. Human embryonic stem cell-derived ACE+CD45-CD34+/- cells are the common yolk sac-like progenitors for not only the endothelium, but also for both primitive and definitive human lymphohematopoietic stem cells [26]. Human angiohematopoiesis initiates from an ACE-hemangioblastic progenitor of primitive and definitive hematopoiesis under the functional activities of the local RAS [26]. Sinka et al. [24] searched for the presence of ACE in the earliest pre-aorta-gonad-mesonephros stages of human intraembryonic angiohematopoiesis. At the earliest stages of human development, hematopoietic potential in the splanchnopleura is restricted to emerging CD34-ACE+ precursors. ACE expression has functions in the maintenance of embryonic hematopoiesis [24]. The results of the present study show that the main RAS components are present in early hematopoietic stem cells and progenitors as well as BM stem, progenitor, and precursor cells. Therefore, manipulation of RAS action could be an important strategy for the expansion of multipotent hematopoietic progenitors during hematopoietic stem cell-related management procedures [46].

The development of MM depends upon deregulation in a complex series of neoplastic pathobiological events. The local tissue RAS influences tumor growth and metastases in an autocrine and paracrine fashion, via the modulation of numerous carcinogenic events such as angiogenesis, apoptosis, cellular proliferation, immune responses, cell signaling, and extracellular matrix formation. Potential manipulation of the local RAS with many enzymes, peptides, and feedback mechanisms can even represent a therapeutic target for the clinical management of cancer [34,47,48]. Our findings about the CD34+MM hematopoietic cells, which locally express critical RAS components in the myeloma-related progenitor cells, could be a starting point for future studies functions on the immunobiology of MM and processing of autologous cells during the formation of transplantation treatment protocols. 

Future experimental and clinical studies are needed to elucidate the puzzling functions of local tissue RASs, including the local BM RAS. These efforts should focus on dissecting local RAS interactions with the complicated pathobiological characteristics of neoplastic disorders and on manipulating autocrine-paracrine-intracrine systems for better clinical management of patients with hematological neoplastic disorders.

## CONFLICT OF INTEREST STATEMENT

The authors of this paper have no conflicts of interest, including specific financial interests, relationships, and/or affiliations relevant to the subject matter or materials included.

## Figures and Tables

**Table 1:**
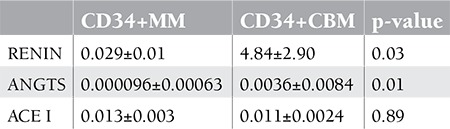
Relative expression levels of RENIN, ANGTS, and ACE I genes compared to CD34+ control bone marrow cells.
